# Npac Regulates Pre-mRNA Splicing in Mouse Embryonic Stem Cells

**DOI:** 10.3390/ijms251910396

**Published:** 2024-09-27

**Authors:** Yiwei Qian, Ying Ye, Wensheng Zhang, Qiang Wu

**Affiliations:** 1 Faculty of Chinese Medicine, Macau University of Science and Technology, Macau, China; 2109853ecw30001@student.must.edu.mo; 2 State Key Laboratory of Quality Research in Chinese Medicines, Macau University of Science and Technology, Macau, China; 3 Cam-Su Genomic Resource Center, Medical College of Soochow University, Suzhou 215123, China

**Keywords:** Npac, RNA splicing, transcription elongation, mouse embryonic stem cells (mESCs)

## Abstract

As a reader of tri-methylated lysine 36 on histone H3 (H3K36me3), Npac has been shown to have a significant role in gene transcription elongation. However, its potential implication in RNA splicing remains unknown. Here, we characterized the phenotypes of *Npac* knockout in mES cells. We discovered that loss of Npac disrupts pluripotency and identity in mESCs. We also found that Npac is associated with many cellular activities, including cell proliferation, differentiation, and transcription regulation. Notably, we uncovered that Npac is associated with RNA splicing machinery. Furthermore, we found that Npac regulates alternative splicing through its interaction with the splicing factors, including Srsf1. Our research thus highlights the important role of Npac in maintaining ESC identity through the regulation of pre-mRNA splicing.

## 1. Introduction

Embryonic stem cells (ESCs) are a type of pluripotent stem cells that are isolated from the inner cell mass (ICM) of preimplantation blastocysts. These cells have the ability for indefinite self-renewal and the capacity to differentiate into cells of all three germ layers: ectoderm, mesoderm, and endoderm, thus proving nearly unlimited cell resources for biomedical research and clinical applications [1,2]. The pluripotency of ESCs is regulated by a cadre of core transcription factors, notably Oct4, Sox2, and Nanog. These factors work in concert to activate genes that promote pluripotency while repressing differentiation signals [3]. Mouse embryonic stem cells (mESCs) are invaluable in developmental biology, offering unparalleled insights into early mammalian development through their superior differentiation potential compared to other mammalian ESCs, including those from humans. Traditionally, mESCs are regarded as representative of the naïve state, while human ESCs are categorized as primed. The naïve state of mESCs is associated with a higher differentiation potential. This capability enables in-depth studies of early cellular differentiation and organogenesis, enhancing our understanding of complex genetic and cellular interactions critical for early embryogenesis [4].

Alternative splicing (AS) epitomizes a vital post-transcriptional regulatory mechanism that significantly augments the proteomic diversity across vertebrates and serves as a pivotal evolutionary hallmark of complex life forms [5]. In eukaryotic cells, the processing of initial transcripts involves a critical mechanism performed by a specialized molecular assembly known as the spliceosome. This complex, consisting of both proteins and RNA components, meticulously coordinates the accurate removal of introns from precursor messenger RNA (pre-mRNA) [6]. Major spliceosomes, which consist of a coordinated assembly of five small nuclear ribonucleoproteins (snRNPs): U1, U2, U4, U5, and U6. The spliceosome undergoes a series of assembly and disassembly steps, each regulated by specific small nuclear RNAs (snRNAs) and associated proteins, ensuring that only correctly spliced mRNA is produced [7]. These entities operate in a dynamic and sequential fashion to ensure the fidelity of splicing events, thereby safeguarding the integrity of the resulting mRNA transcripts. Research has demonstrated that alternative splicing plays an essential role in determining cell fate. For instance, TOBF1 modulates mESC cell fate by regulating alternative splicing of pluripotency genes [8]. Similarly, as an RNA-binding protein, ZFP207 shapes ESC identity through its influence on networks of alternative splicing [9]. Furthermore, increasing evidence suggests that aberrations in splicing patterns are linked to a variety of diseases, including cancer and neurodegenerative disorders [10]. Hence, therapeutic strategies aimed at rectifying these splicing errors are emerging as a new frontier in medical research and innovation [11].

Npac (also named as NP60 or Glyr1), is characterized by its PWWP domain, which can specifically bind to the tri-methylated lysine 36 on histone H3 (H3K36me3) [12], a post-translational modification with significant implications for chromatin architecture and gene regulation. Our research group has utilized ChIP-seq experiments to demonstrate that Npac significantly colocalizes with H3K36me3 within the chromatin of actively transcribed genes in mESCs [13]. The synchronization of transcription and splicing indicates a convergence of cellular processes, linking these mechanisms both temporally and spatially. This suggests a complex interplay where chromatin configuration, the progression of transcription, and the accuracy of splicing are inter-connected [14]. Histone modifications along gene sequences are crucial in the regulation of alternative splicing, as they guide the recruitment of adaptor proteins that recognize specific histone marks. These proteins facilitate the association of splicing factors with the pre-mRNA [15]. For instance, CHD1, a reader of H3K4me3, interacts with the SF3a subcomplex of the U2 snRNP, enhancing pre-mRNA splicing efficiency [16]. Conversely, BS69, which specifically recognizes H3.3K36Me3, opposes the function of the U5 snRNP protein, predominantly encouraging intron retention [17]. Given the strategic position of H3K36me3 at the interface between transcriptional elongation and RNA splicing, we speculate that Npac could serve as a critical regulator within this nexus. Though our previous investigations have shed considerable light on the role of Npac in transcriptional elongation, the involvement of Npac in RNA splicing and the detailed mechanisms underlying this role remain to be elucidated.

Here, we demonstrate that Npac plays a critical role in regulating the identity of mESCs. Our findings reveal that the knockout of *Npac* not only compromises pluripotency but also facilitates differentiation of mESCs. We demonstrate that Npac significantly regulates the transcriptional elongation of pluripotency-associated genes and plays a vital role in the regulation of alternative splicing. Importantly, our data show that Npac can interact with the splicing factor Srsf1, suggesting that Npac modulates splicing activity through this interaction. In summary, our findings on the function of Npac in mESCs reveal the multifunctional nature of this protein, indicating that Npac acts as a regulatory nexus for the proper recruitment of splicing factors. This lays the groundwork for dissecting the complex gene regulatory networks underpinning cell fate determination.

## 2. Results

### 2.1. Loss of Npac Disrupts Pluripotency and Identity in mESCs

**Figure 1:**
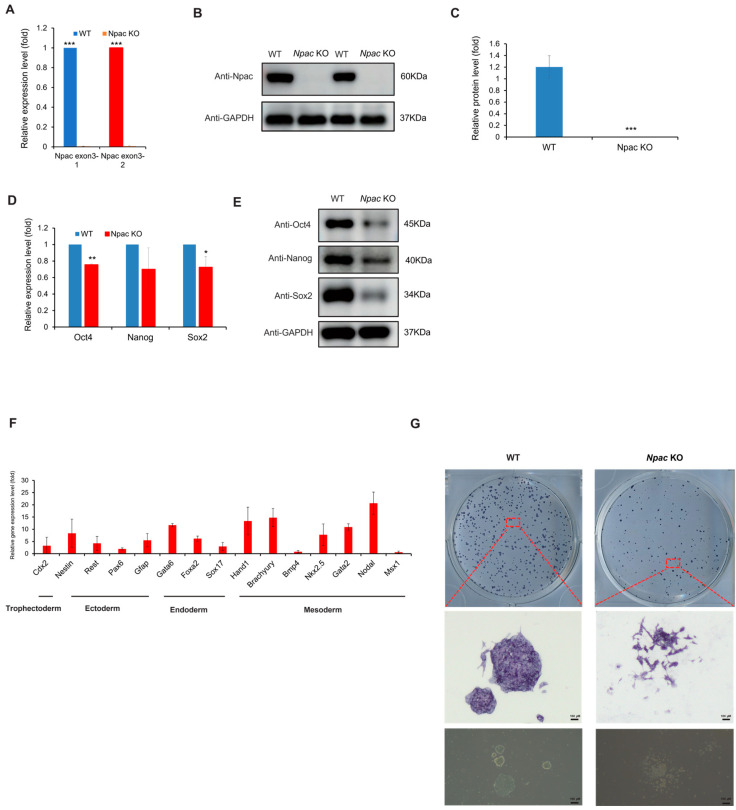
The knockout of *Npac* results in pluripotency defects. (**A**) RT-qPCR was conducted to measure the *Npac* gene expression. (**B**) Western blot was employed to examine the expression levels of Npac protein. (**C**) Protein quantification was carried out. (**D**) RT-qPCR was conducted to evaluate the expression of pluripotent genes. (**E**) Western blot was utilized to examine pluripotent protein levels. (**F**) RT-qPCR was performed to analyze the expression of the lineage-specific marker genes. (**G**) Cells were plated and subjected to AP staining on day 6 post-plating. * *p* < 0.05, ** *p* < 0.01, *** *p* < 0.001.

In our previous studies, we constructed a knockdown model for Npac, revealing that Npac was a novel pluripotency regulator in mESCs [13]. To further investigate the essential role of Npac in mESCs, we first generated *Npac* knockout mESCs using CRISPR/Cas9 technology. To confirm the successful knockout of *Npac* in mESCs, we first employed quantitative PCR (qPCR) and Western blot analysis to detect the expression of Npac at the gene and protein levels, respectively. The results indicated that *Npac* was successfully knocked out (KO) in E14 cells. Our qPCR analysis showed a complete loss of *Npac* mRNA expression, while Western blot confirmed the absence of Npac protein in the KO cells (both *Npac* KO clone 1 and clone 2) (Figure 1A–C). We found that, starting from the first day of cell recovery and continuing with regular cell culture and passaging, significant changes in the expression of certain differentiation genes were observed approximately 2–3 weeks post-recovery. Given the consistency between the clones, we chose to focus our validation efforts on one clone to streamline the experimental process and reduce redundancy. We then proceeded to examine the expression of key pluripotency genes following the knockout of *Npac*. A significant reduction in *Oct4* and *Sox2* mRNA levels was observed, and this was consistent with the decreased protein levels of Oct4, Nanog, and Sox2 in *Npac* KO cells (Figure 1D,E). These findings were consistent with our previous research discoveries and affirmed that Npac plays a critical role in the maintenance of ESC pluripotency. In addition, we observed an upregulation in the expression levels of several lineage-specific marker genes in the *Npac* KO cells. Specifically, we detected an increase in the expression of trophoblast ectodermal markers, such as *Cdx2*. We also observed higher levels of ectodermal markers, including *Nestin*, Rest, and *Gfap*. Additionally, the expression of endodermal markers, such as *Foxa2*, *Sox17*, and Gata6 was elevated. Moreover, we found increased expression of mesodermal markers, including Brachyury, Hand1, Nkx2.5, Gata2, and *Nodal* (Figure 1F). It is noteworthy that not all lineage-specific genes were upregulated, because we observed a decrease in the expression of *Bmp4* and *Msx1*. Morphologically, significant changes were observed in the *Npac* KO cells. Specifically, *Npac* KO cells demonstrated increased cell spreading and a loss of tightly packed colonies, in contrast to the typical compact and rounded morphology of wild-type (WT) cells. Although there were no changes in staining intensity, which was a positive indicator of the ESC marker, alkaline phosphatase activity (Figure 1G). In conclusion, our observations further indicated that Npac is essential for the maintenance of pluripotency in mESCs.

We next explored the function of Npac in lineage determination by evaluating the spontaneous differentiation capacity of *Npac* KO cells into embryoid bodies (EBs), which mimic early stages of mouse embryogenesis. We observed that *Npac* KO cells retain the ability to form EBs. However, the EBs formed by *Npac* KO cells were smaller in size compared to those formed by WT cells (Figure S1A). The size reduction suggests potential intrinsic differences in the differentiation processes between the WT and KO cells, underscoring the possible role of Npac in regulating cellular differentiation. Smaller EBs in *Npac* KO cells indicate an impaired capacity for proper differentiation, reduced proliferative potential, and potentially disrupted signaling pathways critical for differentiation. We observed that the expression of the pluripotency genes *Oct4* and *Sox2* was slightly decreased in comparison to the WT cells, though the difference was not statistically significant. Concurrently, we observed that the expression levels of *Nanog* showed no significant differences on days 2, 4, and 6 except day 8 (Figure S1B). Furthermore, the expression of *Brachyury*, *Msx1*, and *Bmp4* in *Npac* KO EBs was reduced during differentiation, which indicates that the knockout of *Npac* impairs mesodermal specification (Figure S1D). However, we noted that following the knockout, there was an increase in the expression of markers associated with both the ectoderm and endoderm by day 4. This suggests that *Npac* knockout may enhance the specification of lineages within the ectodermal and endodermal layers (Figure S1C,E). These observations indicate that Npac plays a complex role in regulating lineage commitment during the early stages of embryonic development.

### 2.2. Transcriptomic Changes Induced by Npac Knockout

**Figure 2:**
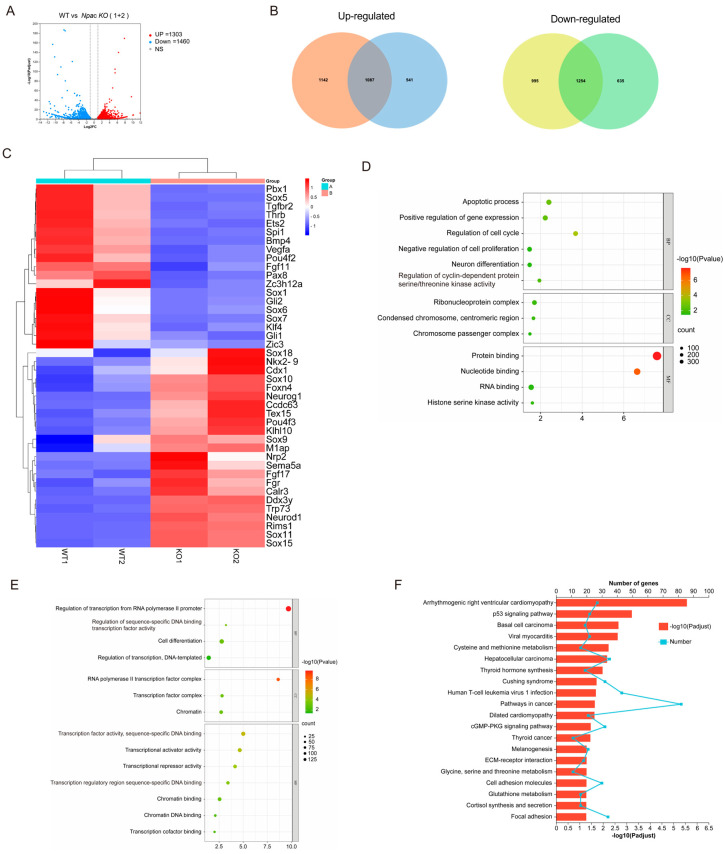
Analysis of RNA-seq results following *Npac* knockout. (**A**) Visualization of RNA-seq results with Volcano plot. (**B**) Venn diagram of differentially expressed genes. (**C**) Visualization of differentially expressed genes with Heatmap. (**D**) GO enrichment for up-regulated genes. (**E**) GO enrichment for down-regulated genes. (**F**) KEGG pathway enrichment analysis of the differentially expressed genes.

To further elucidate the role of Npac in the pluripotency of mESCs, we analyzed the transcriptomic response to *Npac* knockout and found that 1310 genes were significantly up-regulated and 1460 genes were down-regulated based on specific thresholds for fold-change and *p*-value (Figure 2A). Our RNA sequencing (RNA-seq) analysis revealed that in the knockout cell lines KO1 and KO2 mESCs, there were 2229 and 1628 genes up-regulated, and 2249 and 1889 genes down-regulated, respectively (Figure 2B). We then identified differentially expressed genes (DEGs) whose expression levels significantly differ between the WT ES cells and *Npac* KO cells (a fold change > 1.5 and *p* value < 0.05 were set as the thresholds for significantly differential expression). Notably, our analysis of DEGs revealed that genes associated with ESC pluripotency and cellular differentiation exhibited both up-regulation and downregulation following *Npac* knockout. Among these, we observed a down-regulation of pluripotency genes including *Bmp4*, *Klf4*, and *Zic3* and up-regulation of developmental genes including *Sox10*, *Neurod1*, and *Sox15* (Figure 2C). This finding was consistent with our previous RT-qPCR results. Thus, these observations validate the accuracy of our sequencing data. Gene ontology (GO) analysis of commonly up-regulated genes in biological processes revealed shared functions, including cell proliferation, apoptosis, cell cycle, and neuron differentiation (Figure 2D). Functions of commonly down-regulated genes included cell differentiation and transcription factors as well as associations with chromatin regulation (Figure 2E). Enrichment analysis of upregulated genes in KEGG pathways predominantly highlighted the p53 signaling pathway (Figure 2F).

### 2.3. Potential Effects of Npac Knockout on Cellular Proliferation and Apoptosis

**Figure 3:**
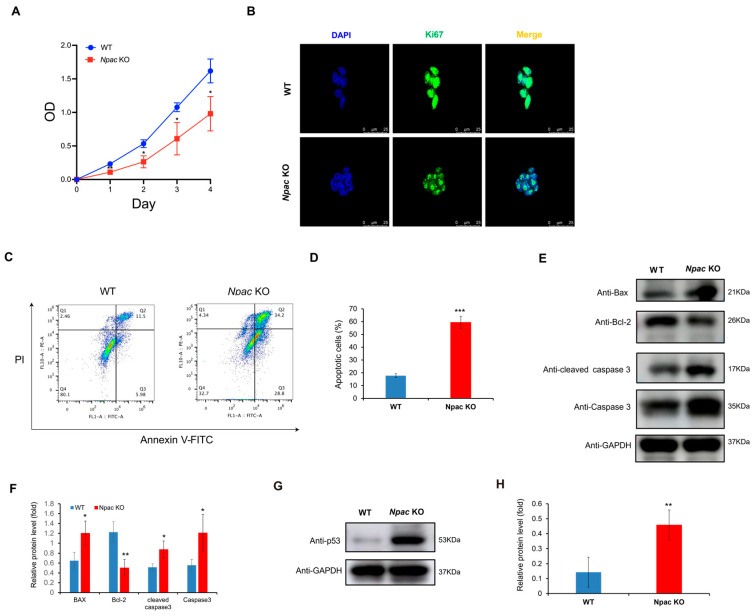
Promotion of apoptosis following Npac knockout. (**A**) Cell proliferation was assessed using the MTT assay. (**B**) Ki67 expression was detected via immunofluorescence. (**C**) Apoptosis induced by Npac knockout was evaluated using flow cytometric analysis with Annexin V staining. (**D**) Apoptotic cells were analyzed by FlowJo, with the results displayed as dot plots. (**E**) The expression of apoptotic proteins was detected by Western blot. (**F**) Quantification of the apoptotic proteins. (**G**) The expression of p53 protein was detected by Western blot. (**H**) Quantification of the p53 protein was conducted. * *p* < 0.05, ** *p* < 0.01, and *** *p* < 0.001.

Based on RNA-seq data, we found that a multitude of genes associated with cell death and apoptosis were up-regulated in the absence of Npac. Based on RNA-seq data, we found that a multitude of genes associated with cell death and apoptosis were up-regulated in the absence of Npac. These genes include *IFITM3*, *BTG2*, and *IFITM1* (negative regulation of cell proliferation); *STEAP3*, *CSRNP3*, and *CASP3* (cell apoptosis). Among these genes, special attention should be paid to *CASP3*, which refers to *Caspase 3*. They represent the same gene and its expression product. We utilized MTT assays to further reveal a significant reduction in cell proliferation in *Npac* KO cells compared to WT cells (Figure 3A). This finding was supported by immunofluorescence assays, which showed diminished expression of the cell proliferation marker Ki-67 in KO cells (Figure 3B). These results collectively suggest that Npac deficiency may promote cell death. Our further flow cytometry analysis revealed that apoptosis was significantly elevated in *Npac* KO cells when compared with their WT cells (Figure 3C,D). Our Western blot analysis corroborated these findings, demonstrating an increase in the expression of pro-apoptotic proteins Bax, Caspase-3, and Cleaved caspase-3, and a decrease in the antiapoptotic protein Bcl-2 (Figure 3E,F).

Interestingly, based on KEGG pathway analysis, we identified a significant enrichment of the p53 pathway, which is known to trigger apoptosis, senescence, and cell cycle arrest in response to cellular stress, thereby preventing the transmission of genetic defects [18,19]. Validation via Western blot confirmed a pronounced increase in p53 protein levels in *Npac* KO cells (Figure 3G,H). Given the established role of the p53 pathway in inducing apoptosis, the observed increase in apoptotic activity in *Npac* KO cells is likely attributable to the activation of this pathway. These findings underscore the critical role of p53 activation in the apoptotic response triggered by a deficiency in Npac.

### 2.4. Knockout of Npac Results in Transcriptional Elongation Defect

Based on our previous research, Npac plays an important role in regulating transcriptional elongation of pluripotency genes in mESCs [13]. Consequently, our current research focused on *Npac* KO models. To further understand the impact of Npac on transcriptional processes, we conducted an elongation rate recovery assay by using 5,6-Dichloro-1-β-D-ribofuranosylbenzimidazole (DRB) as an inhibitor of transcription elongation [20]. This was followed using quantitative RT-qPCR to measure the transcription elongation recovery of specific genes at various positions after the release of an elongation block (Figure S2A,D). We found that transcriptional output of *Nanog* exon 1 and *Tet1* exon 1 closely correlated with the WT cells after the release of the elongation block in Npac KO cells (Figure S2B,E). Conversely, a notable decline in transcriptional recovery was observed at downstream positions, specifically in the exon 4 region of *Nanog* (Figure S2C). Additionally, transcription levels at exon 11 of *Tet1* were subtle diminution relative to control conditions (Figure S2F). These findings affirmed that the loss of Npac can result in transcriptional elongation defects of the pluripotency genes *Nanog* and *Tet1*.

### 2.5. Npac Regulates Alternative Splicing Events in mESCs

Alternative splicing plays a pivotal role in the regulation of gene expression and has become a focal point of interest in genomic research. With the advancements and maturation of sequencing technologies, RNA-seq has emerged as an indispensable tool for in-depth studies of alternative splicing [21]. In our research, we utilized RNA-seq followed by the application of a multivariate analysis program designed for transcript splicing [22] to investigate the spectrum of splicing events following *Npac* knockout.

**Figure 4:**
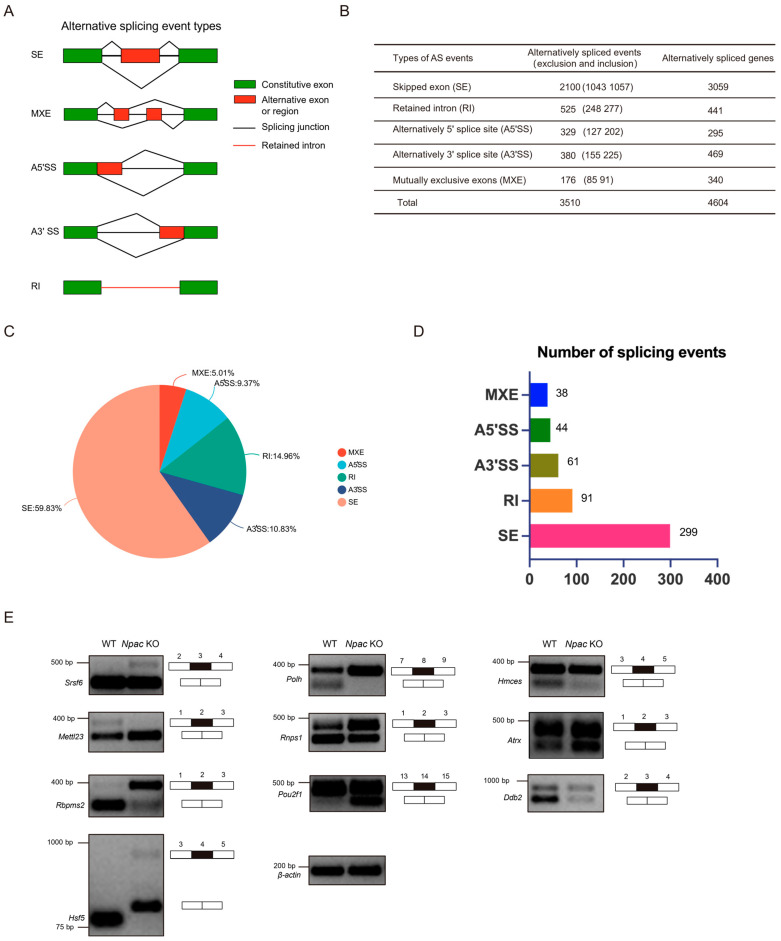
Npac regulates alternative splicing. (**A**) The schematic diagrams illustrate the five principal types of alternative splicing: SE (skipped exons), MXE (mutually exclusive exons), A5′SS, and A3′SS (alternative 5′ and 3′ splice sites, respectively), and RI (retained introns). (**B**) Analysis of RNA-seq data via the rMATs tool enabled the statistical characterization of distinct alternative splicing events and the identification of associated genes in E14 cells. (**C**) A pie chart shows the proportionate distribution of the five categories of splicing alterations induced by *Npac* knockout. (**D**) Utilizing the screening criteria (FDR < 0.05, *p* < 0.05), we initially identified specific alternative splicing events. (**E**) Validation of alternative splicing events in WT and KO E14 cells via quantitative PCR analysis.

Alternative splicing events can be classified into five main types (Figure 4A). We performed a consolidated analysis of the sequencing results from two independent samples (Figure 4B). We discovered that exon skipping (59.83%) was the most prevalent type of alternative splicing (Figure 4C). Following our screening criteria (FDR < 0.05, *p* < 0.05), we initially identified 533 significantly different splicing events, of which exon skipping accounted for 299 events (Figure 4D). Subsequently, genes exhibiting differential alternative splicing were subjected to GO analysis using the DAVID website (https://david.ncifcrf.gov). The analysis of genes undergoing exon skipping revealed that chromatin-related functions were significantly implicated in the biological processes category, including chromatin structure and histone modifications (Figure S3A). In the molecular function category, nucleotide binding was the second most enriched function. Notably, an overrepresentation of protein serine/threonine/tyrosine kinase activity highlighted a significant and previously unrecognized role of Npac in protein functionality. Unexpectedly, genes affected by alterations in A5′ splice sites (A5′SS) were closely linked to RNA splicing, with RNA binding emerging as the most enriched molecular function (Figure S3B). To validate the alternative splicing events identified by the rMATs program, we designed primer pairs capable of detecting specific splicing events through semiquantitative reverse transcription PCR. Specifically, we randomly validated Npac-mediated differentially spliced exons (DSEs) in WT and KO mESCs (Figure 4E). These validations included genes involved in splicing regulation, such as *Srsf6*, Rnps1, and *Rbpms2*; DNA repair mechanisms like *Polh*, *Hmces*, and *Ddb2*; chromatin remodeling processes represented by *Atrx*; transcriptional regulation via *Pou2f1*; RNA/DNA methylation and modification processes, such as *Mettl23*; and the stress response mediated by *Hsf5*. Overall, these data suggest that *Npac* KO can induce aberrant alternative splicing patterns, strongly suggesting a regulatory role of Npac in alternative splicing.

### 2.6. Npac Interacts with Splicing Factors

To uncover how Npac is involved in RNA splicing in mESCs, we utilized the STRING database to generate a protein-protein interaction (PPI) network that includes DEGs (Figure S4). Notably, the critical role of Sf3b3 in pre-mRNA splicing merits focused attention. Sf3b3 is a component of the SF3b complex and serves as an intrinsic part of the functional U2 snRNP [23]. Consequently, this also implies that Npac may play a significant role in the regulation of splicing. To further elucidate the role of Npac, we processed nuclear extracts from E14 cells and employed nonspecific IgG as a negative control. We subsequently performed affinity purification using a specific anti-Npac antibody. This approach enabled us to isolate proteins that interact with Npac, which were then analyzed using liquid chromatography-tandem mass spectrometry (LC-MS/MS) (Figure S5A). Additionally, silver staining of these proteins revealed the presence of nine unique proteins, identified in the experiment (Figure S5B). Interestingly, all these proteins are associated with RNA splicing. Core splicing factors, such as Srsf1, which are essential for both constitutive and alternative splicing, were detected alongside crucial spliceosomal components such as U2af2. This finding suggests the direct involvement of Npac in the assembly and functioning of the spliceosome. Additionally, the detection of Hnrnpk, known to participate in the modulation of alternative splicing. The presence of other significant proteins in the complex, including Nucleolin, Fus, Ddx5, L1td1, Sfpq, and Refbp2, further supports the extensive network of interactions mediated by Npac within RNA splicing pathways. Interactions between them have been identified via the STRING database (Figure S5C), indicating that the protein interaction network may play a pivotal role in the regulation of splicing by Npac.

**Figure 5:**
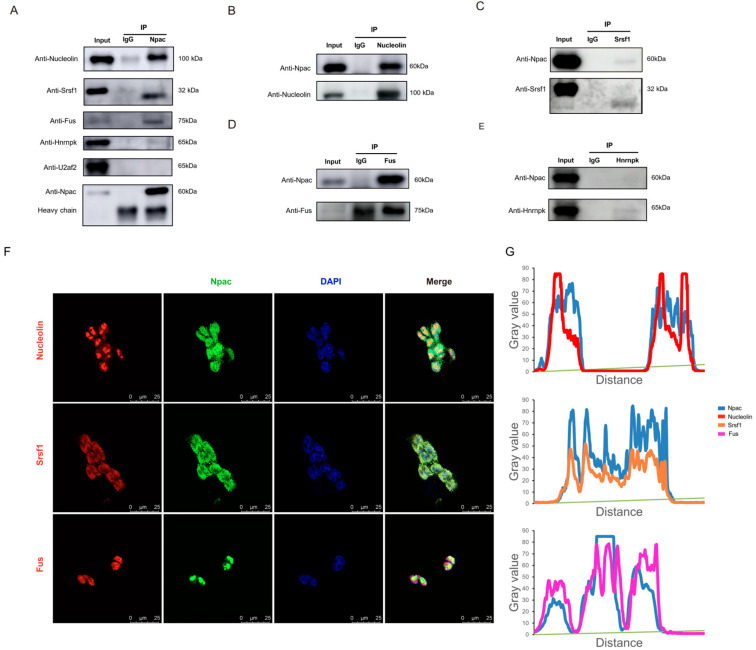
Npac interacts with splicing factors-associated proteins. (**A**) Incubation of anti-Npac with cell lysis from E14 cells enabled the pulldown of Nucleolin, Srsf1, Fus, U2af2, and hnrnpk. (**B**–**E**) Immunoprecipitation with anti-Nucleolin, anti-Srsf1, anti-Fus, anti-hnrnpk or control IgG was performed in each fraction, followed by immunoblotting for indicated proteins. (**F**) The localization of Npac (green) and indicated proteins (red) in mESC cells was analyzed by immunofluorescence. Cell nuclei were labeled by DAPI (blue) staining. Scale bar: 25 µm. (**G**) The fluorescence intensity profiles for Npac, Srsf1, Nucleolin, and Fus were quantitatively assessed along a designated line within the composite image using ImageJ software version 1.54d.

Subsequently, we validated the interactions between Npac and splicing proteins via coimmunoprecipitation (Co-IP). Our assays confirmed interactions between Npac and Srsf1, Nucleolin, Fus (Figure 5A–D). However, we did not observe any interaction between Npac and U2af2 or Hnrnpk (Figure 5A,E). Immunofluorescence staining assays further supported these findings by demonstrating the nuclear colocalization of Npac with Srsf1, Nucleolin, and Fus (Figure 5F,G). Moreover, RT-qPCR and Western blot analyses of Npac KO cells revealed decreased expression levels of Srsf1, Nucleolin, and Fus (Figure S6). It is important to note that SRSF proteins function as essential transacting factors that facilitate the splicing of pre-mRNA and are critical for the assembly of spliceosomal components, as well as for regulating splicing fidelity and efficiency [24]. Hence, Npac appears to regulate alternative splicing through its interactions with splicing factors Srsf1.

### 2.7. Npac Interacts with Components of Spliceosomal Machinery

**Figure 6:**
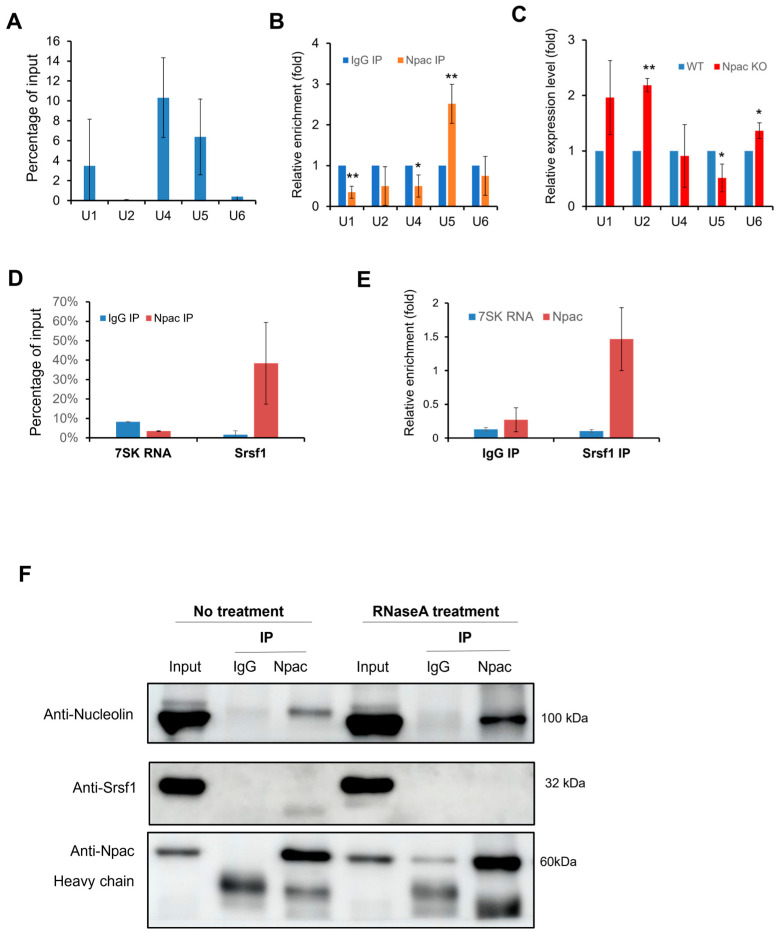
Npac interacts with components of spliceosomal machinery. (**A**) RIP with Npac antibody. The U SnRNA percentage of input detected by RT-qPCR. (**B**) Native RIP assays were performed with both Npac and IgG antibodies to assess the relative enrichment of the specified U snRNAs in Npac IP compared with IgG controls, with *β-actin* mRNA serving as a reference for normalization. (**C**) RT-qPCR was applied to detect the five spliceosomal snRNAs in E14 cells. (**D**) Native RIP assay combined with RT-qPCR was used to detect *Srsf1* and *7SK* RNA isolated by Npac antibody or normal IgG in E14 cells. (**E**) Native RIP followed by RT-qPCR detecting *Npac* and *7SK* RNA retrieved by Srsf1 antibody or by normal IgG in E14 cells. (**F**) Interactions between indicated proteins and Npac were confirmed by coimmunoprecipitation and Western blotting analyses. Samples were treated with or without RNase A. * *p* < 0.05, ** *p* < 0.01.

The interaction between Npac and the splicing factor Srsf1 may link it to the spliceosome, as Srsf1 is directly involved in the assembly of the spliceosome [25]. This interaction suggests that Npac is broadly involved in the components of the spliceosome, particularly the small nuclear ribonucleoproteins (snRNPs). Consequently, we proceeded with RNA immunoprecipitation (RIP) experiments to explore this hypothesis further (Figure S7A). In native RIP assays, Npac specifically coprecipitated with *U1*, *U4*, and *U5* snRNAs, showing a notable enrichment of *U5* snRNA compared to the IgG control (Figure 6A,B). Additionally, crosslinked RIP assays, which stabilize RNA-protein interactions using formaldehyde, revealed increased levels of *U4* and *U5* snRNAs in the presence of Npac, relative to control (Figure S7B). Interestingly, following the knockout of *Npac* in E14 cells, only the expression of *U5* snRNA was observed to decrease (Figure 6C). These findings suggest that Npac may interact with *U5* snRNA.

Simultaneously, we utilized Native RIP assays to verify the interactions between Npac and previously identified splicing factors. We included *7SK* RNA, a nuclear long noncoding RNA (lncRNA) known for its involvement in transcription initiation, to serve as a negative control in our experiments. We found that *Srsf1* exhibited the highest level of enrichment (Figure S7C). Further native RIP assays confirmed the interaction between Npac and Srsf1 (Figure 6D,E). To elucidate the mechanisms by which Npac influences the assembly process of the spliceosome, we sought to verify whether the interaction between Npac and the Srsf1 is mediated via RNA. Subsequently, we treated E14 cell nuclear extracts with RNase to eliminate RNA-mediated interactions and then performed Co-IP. Post-RNase treatment, the expression of Srsf1 was reduced. Interestingly, we also assessed that the expression of the Nucleolin protein was increased (Figure 6F). These findings imply that interaction between Npac and Srsf1 could be contingent upon the presence of snRNA. Overall, these findings support the potential role of Npac in regulating RNA splicing.

## 3. Discussion

The core transcriptional circuitry of ESCs, crucial for maintaining pluripotency, is centered around the triad of transcription factors: Oct4, Sox2, and Nanog. These factors collaboratively activate genes that promote pluripotency and repress genes specific to differentiation. This dynamic is further stabilized by intricate feedback loops and multifaceted crosstalk among various regulatory strata, which collectively enhance the resilience of the ESC transcriptional framework [26,27,28,29,30]. We evaluated Npac’s role in early embryonic events by inducing in vitro differentiation of mESCs into the three germ layers through EB formation. Our findings indicate that *Npac* knockout inhibited EB formation and growth yet increased ectodermal and endodermal marker expression while decreasing mesodermal marker expression. These findings suggest that Npac plays a pivotal role in modulating the balance of cell fate decisions, potentially by influencing the activity of key pluripotency factors and their downstream targets.

We have observed that a deficiency in Npac results in transcriptional elongation defects in pluripotency-associated genes, specifically *Nanog* and *Tet1*. Consistent with previous findings [13]. Additionally, it is noteworthy that Tet1 is highly expressed in ESCs and plays a pivotal role in maintaining the transcriptional network essential for pluripotency in these cells [31,32]. These findings collectively highlight the indispensable role of Npac in facilitating transcriptional elongation, reinforcing its importance in the molecular framework of stem cell biology. Furthermore, the intricate relationship between transcription and splicing, often described as co-transcriptional splicing, plays a vital role in ensuring accurate and regulated gene expression in eukaryotic organisms. The process of transcription involves RNA Pol II synthesizing pre-mRNA from DNA, which is immediately followed by splicing. During this subsequent phase, noncoding introns are removed, and coding exons are connected to form mature mRNA [33,34].

Alternative splicing is crucial for gene expression, enabling a single gene to produce multiple protein isoforms. This enhances protein diversity and biological complexity in eukaryotes. This process is essential for development and adaptation, and its dysregulation is linked to various diseases. RNA-seq has transformed our ability to study alternative splicing by identifying novel splicing events and estimating their functional impacts. However, this technique faces challenges, such as assembling full-length transcripts from short reads, which may underestimate splicing complexity [22]. Our study utilized RNA-seq to reveal that exon skipping is the predominant alternative splicing event following *Npac* knockout, a finding we have experimentally validated. The insights gained from manipulating splicing patterns hold therapeutic promise, particularly for disorders like certain cancers and spinal muscular atrophy, underscoring the importance of advancing RNA-seq and bioinformatics tools for an enhanced understanding of splicing dynamics in health and disease [35].

Transacting factors influencing alternative splicing are primarily divided into two groups: serine-arginine-rich splicing factors (SR proteins) and heterogeneous nuclear ribonucleoproteins (hnRNPs), which generally act as activators and inhibitors of splicing, respectively [36]. SR proteins, a highly conserved family of RNA-binding proteins, are crucial for cellular viability and play a pivotal role in spliceosome assembly by stabilizing and orchestrating the splicing of pre-mRNA [37]. These proteins bind to exonic splicing enhancers (ESEs), promoting both constitutive and alternative splicing by recruiting the spliceosome [38,39]. Specifically, they enhance the interaction between U1 snRNP and pre-mRNA, which is crucial for the alignment of U2 auxiliary factor (U2AF) with U2 snRNP at the 3′ splice site. This alignment is a critical step for recognizing and removing introns, and it supports the subsequent linking of exons [25,40,41]. SRSF1, a key member of the SR protein family, is regulated by phosphorylation of its RS domain and plays a pivotal role in both constitutive and alternative RNA splicing [42]. It also facilitates early spliceosome assembly by engaging with the U1 snRNP [25]. Additionally, the RNA-binding protein FUS is engaged in a variety of RNA biosynthesis processes [43] and plays a key role in linking transcription to splicing by mediating the interaction between RNA Pol II and U1 snRNP [44]. Another significant protein, Nucleolin (NCL), is abundant, highly conserved, and multifunctional and has been reported to be associated with the endogenous spliceosome [45]. Our findings reveal that Npac interacts with Srsf1, Nucleolin, and Fus, suggesting that Npac may modulate alternative splicing through these connections. Our RIP experiments further demonstrate that Npac primarily interacts with Srsf1, indicating a regulatory role in splicing. Serine/arginine-rich splicing factor 1 (SRSF1), formerly known as SF2/ASF, is a pivotal RNA-binding protein that plays a critical role in the regulation of splicing. Additionally, it has been demonstrated to be involved in the transcription, stability, and nuclear export of mRNA, as well as in translation, nonsense-mediated mRNA decay (NMD), and sumoylation processes [46]. Recent studies suggest that there may be a link between Srsf1 and the H3K36me3. It has been reported that the H3K36me3 “reader” Psip1/Ledgf, containing a PWWP domain, acts as an adapter that facilitates the recruitment of SRSF1 to chromatin modified by H3K36me3, thereby promoting splicing regulation [47]. Recent studies have shown that alternative splicing significantly impacts the pluripotency of embryonic stem cells and their fate decisions [48]. In our research, we discovered that Npac not only acts as a splicing regulatory factor but also interacts with the core component of the spliceosome, *U5* snRNA. This underscores the role of Npac in regulating splicing. However, we have not yet verified whether Npac directly regulates pluripotency through a splicing mechanism. It remains unclear how Npac acts as an RNA-binding protein (RBP) and regulates RNA splicing, thereby further modulating the cell fate decisions in ES cells. Nonetheless, our observations highlight the intricate role of Npac in the regulation of splicing and underscore its potential as a key player in the post-transcriptional control of gene expression. The complexity of Npac’s function emphasizes the need for further investigation into the molecular mechanisms by which it influences splicing dynamics.

Taken together, we confirm that Npac can serve as a novel alternative RNA splicing regulator. We propose that Npac interacts with multiple splicing factors, such as Srsf1, a key member of the SR protein family, facilitates early spliceosome assembly, and indicates that Npac plays a regulatory role in splicing. Hence, our study reveals Npac as a novel regulator of alternative splicing in mESCs, highlighting its critical role in maintaining pluripotency and influencing cell fate decisions. These findings pave the way for further exploration into the molecular mechanisms governing stem cell biology and may inform future therapeutic strategies targeting splicing dysregulation in diseases. In conclusion, our findings illuminate the multiple roles of Npac in the intricate gene regulatory networks and cell fate decisions of mESCs.

## 4. Materials and Methods

### 4.1. Antibodies

The following commercially available antibodies were used at the indicated concentrations for western blot: Npac (Proteintech, Rosemont, IL, USA. 14833-1-AP, 1:1000), GAPDH (Proteintech, Rosemont, IL, USA. 60004-1-Ig, 1:20,000), Oct4 (Santa Cruz Biotechnology, Dallas, TX, USA. sc-8628, 1:200), Nanog (Santa Cruz Biotechnology, Dallas, TX, USA. sc-134218, 1:200), Sox2 (Santa Cruz Biotechnology, Dallas, TX, USA. sc-365823, 1:1000), SF2/ASF (Santa Cruz Biotechnology, Dallas, TX, USA. sc-33652, 1:100), Nucleolin (Abcam, Cambridge, UK. ab22758, 1:1000), Fus (Santa Cruz Biotechnology, Dallas, TX, USA. sc-47711, 1:1000), Hnrnpk (Santa Cruz Biotechnology, Dallas, TX, USA. sc-28380, 1:5000), U2af2 (Santa Cruz Biotechnology, Dallas, TX, USA. sc-374333, 1:5000), Bax (CST, Danvers, MA, USA. 1:1000), Bcl-2 antibody (CST, Danvers, MA, USA. 4223s, 1:1000), Caspase3 (CST, Danvers, MA, USA. 9662s, 1:1000), Cleaved caspase-3 (CST, Danvers, MA, USA. 9661s, 1:1000), p53 (CST, Danvers, MA, USA. 9282, 1:1000), Goat Anti-Rabbit IgG H&L (HRP) (Beyotime, Shanghai, China. A0208, 1:1000), Goat Anti-Mouse IgG H&L (HRP) (Beyotime, A0192, 1:1000), Donkey Anti-Goat IgG H&L (HRP) (Beyotime, Shanghai, China. A0181, 1:1000). For IF staining, we used Ki67 (CST, Danvers, MA, USA. 9129, 1:400), Alexa Fluor 647-labeled Goat Anti-Mouse IgG (H+L) (Beyotime, Shanghai, China. A0473, 1:500), Alexa Fluor 488-labeled Goat Anti-Rabbit IgG (H+L) (Beyotime, Shanghai, China. A0423, 1:500), and Alexa Fluor 555-labeled Donkey Anti-Rabbit IgG (H+L) (Beyotime, Shanghai, China. A0453, 1:500). Cy3-labeled Donkey Anti-Goat IgG (H+L) (Beyotime, Shanghai, China. A0502, 1:500).

### 4.2. Cell Culture

E14 cells were cultured on 0.1% gelatin-coated tissue culture plates under feeder free culture conditions at 37 °C with 5% CO2 in a humidified incubator. The media composition consists of Gibco Glasgow’s MEM (GMEM), 15% fetal bovine serum (FBS; Gibco, Seoul, Republic of Korea), 1% MEM nonessential amino acids (NEAA; Gibco), 100 mM Sodium Pyruvate (Gibco), 55 mM β mercaptoethanol (Gibco), 1000 U/mL Leukemia inhibitory factor (LIF; Millipore), 1% penicillin/streptomycin (Gibco). The media were replaced every 24 h.

#### 4.2.1. Generation of CRISPR/Cas9 Knockout ESCs

To generate knockout cells, 2 μgof gRNA and 2 μgof Cas9 plasmids were electroporated to ESCs. After 7 days’ selection with 1 μg/mL of puromycin, colonies were picked up for genotyping and confirmed by Sanger sequencing and qPCR analysis. The primers for generating specific gRNA plasmids were shown in the following table.


**Plasmid**

**Primer**
exon3-sgRNA-F1atgCGTCTCaACCGAGCGTTCATAACTCTACATGgttttagagctagaaatagcaagexon3-sgRNA-R1atgCGTCTCgAAACCGAGTCATTGCAATAAGACTCGGTGTTTCGTCCTTTCCACAAGexon3-sgRNA-F2atgCGTCTCaACCGTTGGACAATATACTACCTGTgttttagagctagaaatagcaagexon3-sgRNA-R2atgCGTCTCgAAACGCGTTCATAACTCTACATGTCGGTGTTTCGTCCTTTCCACAAG

#### 4.2.2. EB Assay

Embryoid bodies (EBs) were produced by culturing mouse embryonic stem cells (ESCs) in a nonadherent six-well plate using a full nutrient mixture (GMEM by Gibco, supplemented with 15% fetal bovine serum, 1% nonessential amino acids, 100 mM sodium pyruvate, 55 mM β-mercaptoethanol, and 1% penicillin/streptomycin, all from Gibco) without the addition of LIF. The cell seeding density was maintained at 50,000 cells per well. The culture medium was refreshed twice a day. At predetermined time intervals, the EBs were collected for the purpose of isolating total RNA.

#### 4.2.3. Cell Proliferation

To assess cellular proliferation, an MTT assay was performed. E14 cells were initially plated at a density of 2000 cells per well within a 96-well plate. Over a period extending to 96 h, with assessments at 24-h intervals, the cells underwent treatment with MTT solution for 4 h at each time point. Ultimately, the viability of these cells was quantitatively evaluated by measuring the optical density (OD).

#### 4.2.4. Cell Apoptosis Assay

The apoptosis assay was performed in accordance with the guidelines provided by the manufacturer. Cells were subjected to either knockout or wild-type treatments and maintained under these conditions for 2 days. Following this period, the cells were washed twice using ice-cold PBS. For detection of apoptotic cells, they were subsequently stained with Annexin V FITC and Propidium Iodide. The fluorescence intensity of the stained cells was quantitatively analyzed using a BD FACSAriaTM III flow cytometer, enabling precise evaluation of apoptosis levels.

#### 4.2.5. Alkaline Phosphatase Staining

Alkaline phosphatase (AP) staining was performed utilizing the Alkaline Phosphatase Detection Kit (Beyotime, C3206), strictly following the protocols provided by the manufacturer. Initially, the cells were subjected to three PBS washes and subsequently fixed in 4% paraformaldehyde for a duration of 30 min. After the fixation process, the cells were washed another three times with PBS, each wash lasting approximately 5 min. For the staining process, 1 mL of AP substrate solution, which consisted of 10 μL BCIP and 20 μL NBT in 3 mL of AP buffer, was added to each well of a 6-well plate. The plates were then incubated at room temperature, shielded from light for one hour. To terminate the staining reaction, the wells were rinsed with water and allowed to air dry before proceeding with imaging analysis.

#### 4.2.6. Real-Time PCR and RT-PCR

Total RNA extraction was conducted using the TransZol Up Plus RNA Kit (TransGen, Beijing, China. ER501-01), strictly adhering to the manufacturer’s protocols. Quantitative assessment of RNA concentration and quality was performed using NanoDrop. Subsequently, one microgram of the extracted RNA was used for the synthesis of complementary DNA (cDNA) employing the TransScript^®^ All-in-One First-Strand cDNA Synthesis SuperMix for qPCR (TransGen, Beijing, China. AT341-01), as specified by the manufacturer’s instructions. Reverse transcription quantitative polymerase chain reaction (RT-qPCR) was carried out in triplicate using the PerfectStart^®^ Green qPCR SuperMix (TransGen, Beijing, China. AQ602-21) within a ViiA 7 Real-Time PCR System (Applied Biosystems, Waltham, MA, USA). *β-Actin* served as the reference gene to normalize the RNA input levels. To ensure the specificity of the PCR products, melting curve analysis was conducted following each amplification cycle. The expression levels of the examined genes were determined using the 2^−ΔΔCt^ method, incorporating data from a minimum of three biological replicates. The specific primer sequences used for these qPCR analyses were detailed in Table S1. RT-PCR was performed using the DreamTaq Green Master mix (Vazyme, Nanjing, China. P111-01). The cycling parameters were shown as follows: predenaturation at 95 °C for 3 min, 35 cycles of denaturation at 95 °C for 15 sec, annealing at 60 °C for 15 sec, extension at 72 °C for 60 sec/kb, and complete extension at 72 °C for 5 min. The specific primers used for AS were listed in Table S2.

#### 4.2.7. RNA-Seq and Differential Gene Expression Analysis

RNA-seq library preparation was carried out at Novogene facilities “https://en.novogene.com/ (accessed on 5 June 2023)” and sequenced using the Illumina HiSeq 2500 platform (Illumina, San Diego, CA, USA) as 150-bp pair-ended reads. The clustering of the index-coded samples was performed on a cBot Cluster Generation System using the PE Cluster Kit cBot-HS (Illumina, San Diego, CA, USA). Raw data (raw reads) in FASTQ format were first processed through FASTQ. All downstream analyses were based on clean data with high quality. Reference genome and gene model annotation files were downloaded from the genome website browser (NCBI/UCSC/Ensembl) directly. Paired-end clean reads were mapped to the reference genome using the HISAT 2.2.1 software. Differential expression analysis of two conditions was performed using the edgeR R package. The *p* values were adjusted using the Benjamini and Hochberg methods. An adjusted *p* value < 0.05 and a fold change > 1.5 were set as the thresholds for significantly differential expression. The RNA-seq data has been deposited into the GEO database (accession number GSE274429).

#### 4.2.8. Immunofluorescence Staining

E14 cells were cultured in six wells till 80% confluency. Cells were washed with PBS and fixed with 4% paraformaldehyde for 30 min. The cells were washed 3 times with PBS and treated with 0.1% Triton X-100 at room temperature for 20 min. Blocking was performed using 5% BSA for 1 h at room temperature. Cells were washed and incubated overnight with primary antibody at 4 °C. The samples were incubated with secondary antibody for 1 h in the dark at 4 °C after washing three times with TBST. The nuclei were stained with DAPI for 10 min at room temperature. Finally, the staining signals were observed, and images were captured by confocal microscopy (TCS SP8, Leica, Germany).

#### 4.2.9. Gene Ontology, KEGG and Functional Domain Cluster Analysis

Enrichment analysis of differentially expressed genes was conducted to delineate their involvement in KEGG pathways, as well as to categorize them according to Gene Ontology (GO) terms, encompassing biological processes (BP), molecular functions (MF), and cellular components (CC). This analysis utilized a comprehensive database for annotation, visualization, and integrated discovery (DAVID) (https://david.ncifcrf.gov).

#### 4.2.10. Coimmunoprecipitation (Co-IP)

Confluent cells were harvested and subjected to two washes with ice-cold PBS before resuspension in NP40 cell lysis buffer (50 mM Tris-HCl, pH 8.0, 1 mM EDTA, 150 mM NaCl, 1% NP-40, and 10% glycerol), enriched with a protease inhibitor cocktail (Roche Basel, Switzerland). This mixture was incubated at 4 °C for 60 min. Subsequently, the lysate was centrifuged at 14,000 rpm for 30 min, and the supernatant collected was precleared with Protein A agarose beads (Invitrogen, Waltham, MA, USA) at 4 °C for 2 h. Following pre-clearance, beads precoated with a specific antibody were introduced into the supernatant, and the assembly was incubated at 4 °C for an additional 2 h. After an overnight incubation at 4 °C, the beads were subjected to five sequential washes with NP40 lysis buffer containing protease inhibitors to remove nonspecifically bound proteins. For Western blot analysis, the beads were then mixed with 20 µL of 5× loading dye, and the total volume was adjusted with additional NP40 buffer. The samples were subsequently heated at 70 °C for 45 min to denature the proteins adequately.

#### 4.2.11. Western Blotting

Cells were harvested and subjected to two rounds of washing with ice-cold PBS prior to lysis in RIPA buffer (Abcam, AB156034). The resultant proteins were sepatrated onto a 10% sodium dodecyl sulfate (SDS) polyacrylamide gel. The separated proteins were then electrotransferred onto a methanol-preactivated polyvinylidene fluoride (PVDF) membrane (Millipore, Burlington, MA, USA). The membrane was subsequently incubated with a target-specific primary antibody after blocking. The membrane was washed three times and then incubated with an appropriate horseradish peroxidase (HRP)-conjugated secondary antibody. The chemiluminescent signals were detected using an Amersham ImageQuant 800 under controlled dark conditions, ensuring precise and reproducible quantification of the protein bands.

#### 4.2.12. Transcription Elongation Assay

E14 cells (mES cell line) were exposed to 100 μM of 5,6-Dichloro-1-β-D-ribofuranosylbenzimidazole (DRB) (287891, Sigma, St. Louis, MO, USA) for a duration of 3 h. The cells were rinsed twice with PBS and subsequently incubated in fresh culture medium for variable time intervals (5–60 min). Total RNA was then extracted from the cells. To assess the dynamic changes in transcriptional activity, relative mRNA levels at various regions within the Nanog and Tet1 genes were quantified using RT-qPCR. For normalization of gene expression data, β-actin was employed as an internal control. Detailed sequences of the primers used for RT-qPCR were provided in Supplementary Table S3.

#### 4.2.13. Native RNA Immunoprecipitation (RIP)

A native RIP assay was performed as previously described with some modification [49,50]. Briefly, a total of 1 × 107 cells were washed twice in cold PBS. Resuspend cells in an equal pellet volume of polysome lysis buffer (10× PLB: 1000 mM KCl, 50 mM MgCl_2_, 100 mM HEPES-NaOH pH 7, 5% Nonidet P-40 (NP-40). Before using it, prepare PLB dilution to 1× and add 1 mM dithiothreitol (DTT), 50 units/mL RNase OUT, and EDTA-free Protease Inhibitor Cocktail). After an incubation with specific Ab or control IgG with rotation for 2 h at room temperature. Samples were incubated with 100 μL of protein G magnetic beads overnight at 4 °C, and then washed three times in with 0.5 mL NT-2 buffer (5×: 250 mM Tris-HCl pH 7.4, 750 mM NaCl, 5 mM MgCl_2_, 0.25% NP-40). The beads were resuspended and treated with proteinase K at 55 °C for 30 min. Coprecipitated RNAs were extracted using TRIzol reagent, and then detected by RT-qPCR. The primers used for RT-qPCR following native RIP detecting specific RNA transcripts were shown in Table S4.

#### 4.2.14. Nuclear Extracts (NEs) Preparation

E14 cells were homogenized in Buffer A (10 mM Tris-HCl pH 7.9, 10 mM KCl, 10% glycerol, 1.5 mM MgCl_2_ supplemented with fresh 0.5 mM DTT, protease inhibitors (Roche), and 0.2 mM PMSF) on ice. Nuclei were sedimented by centrifugation (1000× *g*, at 4 °C) and suspended in Buffer C (20 mM Tris-HCl pH 7.9, 1.5 mM MgCl_2_, 0.42 M NaCl, 0.2 mM EDTA, 25% glycerol, 0.5 mM DTT, and protease inhibitors). Soluble nuclear proteins were separated by centrifugation (20,000× *g* for 30 min, at 4 °C) and dialyzed against BC50 buffer (20 mM Tris-HCl pH 7.9, 50 mM KCl, 0.2 mM EDTA, 10% glycerol, 10 mM ß-mercaptoehtanol) for 24 h. Samples were centrifuged at 20,000× *g* for 30 min at 4 °C and the suspernatant was kept at −80 °C. NEs were treated either with RNase A (100 μg/mL) for 15 min or with DNase I (10 U) for 30 min at 37 °C. Then, immunoprecipitations were then performed with samples (with or without treatment) resuspended in standard immunoprecipitation buffer and rotated overnight with beads cross-linked with Npac antibody. Rabbit IgG was used as a negative control. For NE samples from mECSs, immnoprecipitations were performed using either beads cross-linked with SF2/ASF antibody or Nucleolin antibody.

## 5. Statistical Analysis

Statistical analysis of the data was performed using GraphPad Prism 9. The significance was determined using the student’s t-test. Probability values of * *p* < 0.05, ** *p* < 0.01, *** *p* < 0.001 **** *p* < 0.0001 were considered as statistically significant.

## Data Availability

Data is contained within the article and Supplementary Materials.

## Supplementary Materials

The following supporting information can be downloaded at: https://www.mdpi.com/article/10.3390/ijms251910396/s1.
