# Supplementary material for: Npac Regulates Pre-mRNA Splicing in Mouse Embryonic Stem Cells
Source: Int J Mol Sci. 2024 Sep 27;25(19):10396. doi: 10.3390/ijms251910396 (PMC11477393; doi:10.3390/ijms251910396)
Supplement: Supplementary file 1 [file ijms-25-10396-s001.zip › ijms-3199804-supplementary.pdf]

Supplementary Materials for

Npac regulates pre-mRNA splicing in mouse embryonic stem cells

Supplementary Figure

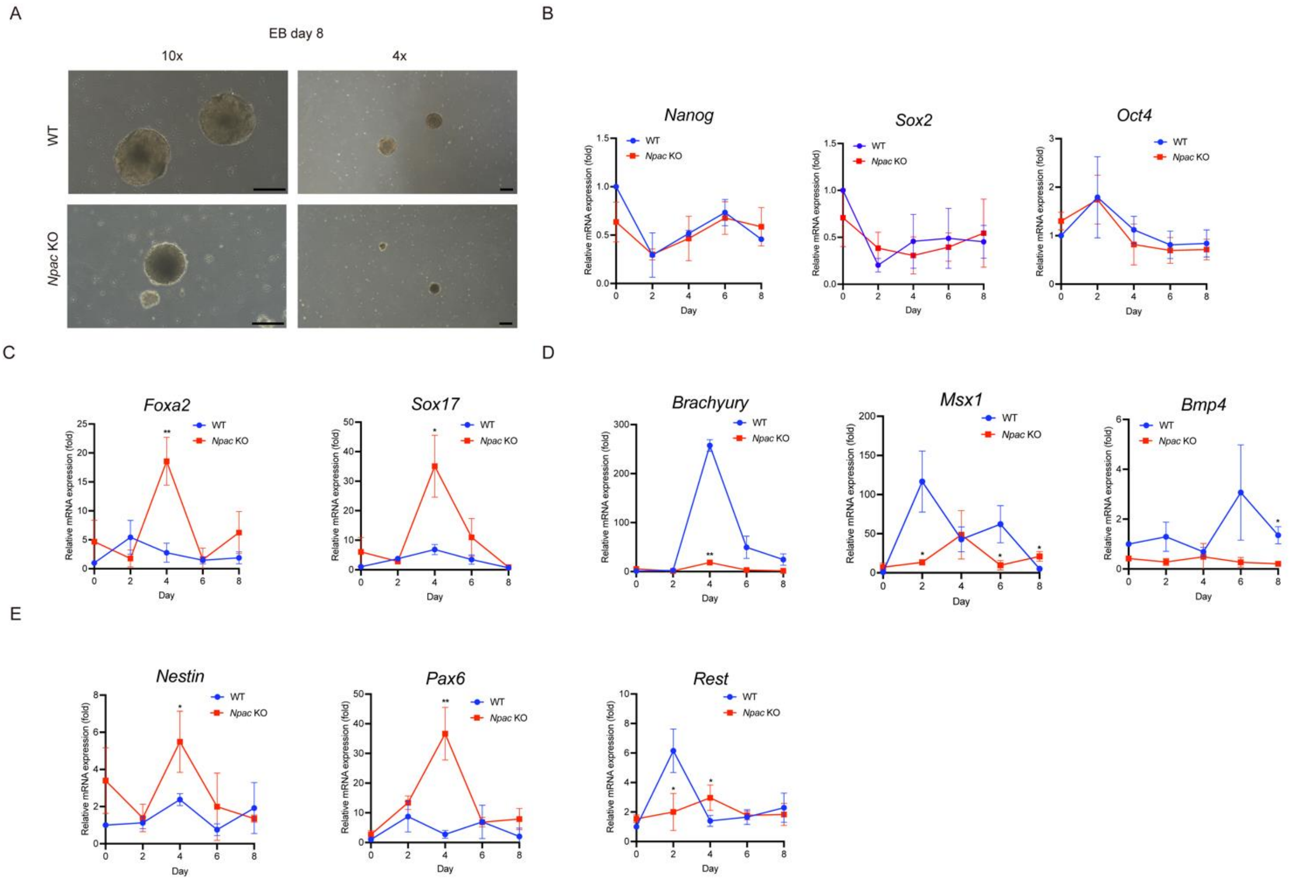

Figure S1. Loss of *Npac* affects differentiation. (A) Bright field images displaying EBs formed from wild-type (WT) and knockout (KO) cells on day 8 of differentiation at 10x (left) and 4x (right) magnifications. Scale bars represent 200 µm. (B-E) RT-qPCR of the pluripotency genes (*Nanog, Oct4 and Sox2*), the endodermal markers (*Foxa2, Sox17*), the mesodermal markers (*Brachyury Msx1 and Bmp4*), and the ectodermal genes (*Nestin, Pax6 and Rest*) in *Npac* KO cells during the progression of EB-mediated differentiation. *$p < 0.05$, **$p< 0.01$, ***$p < 0.001$.

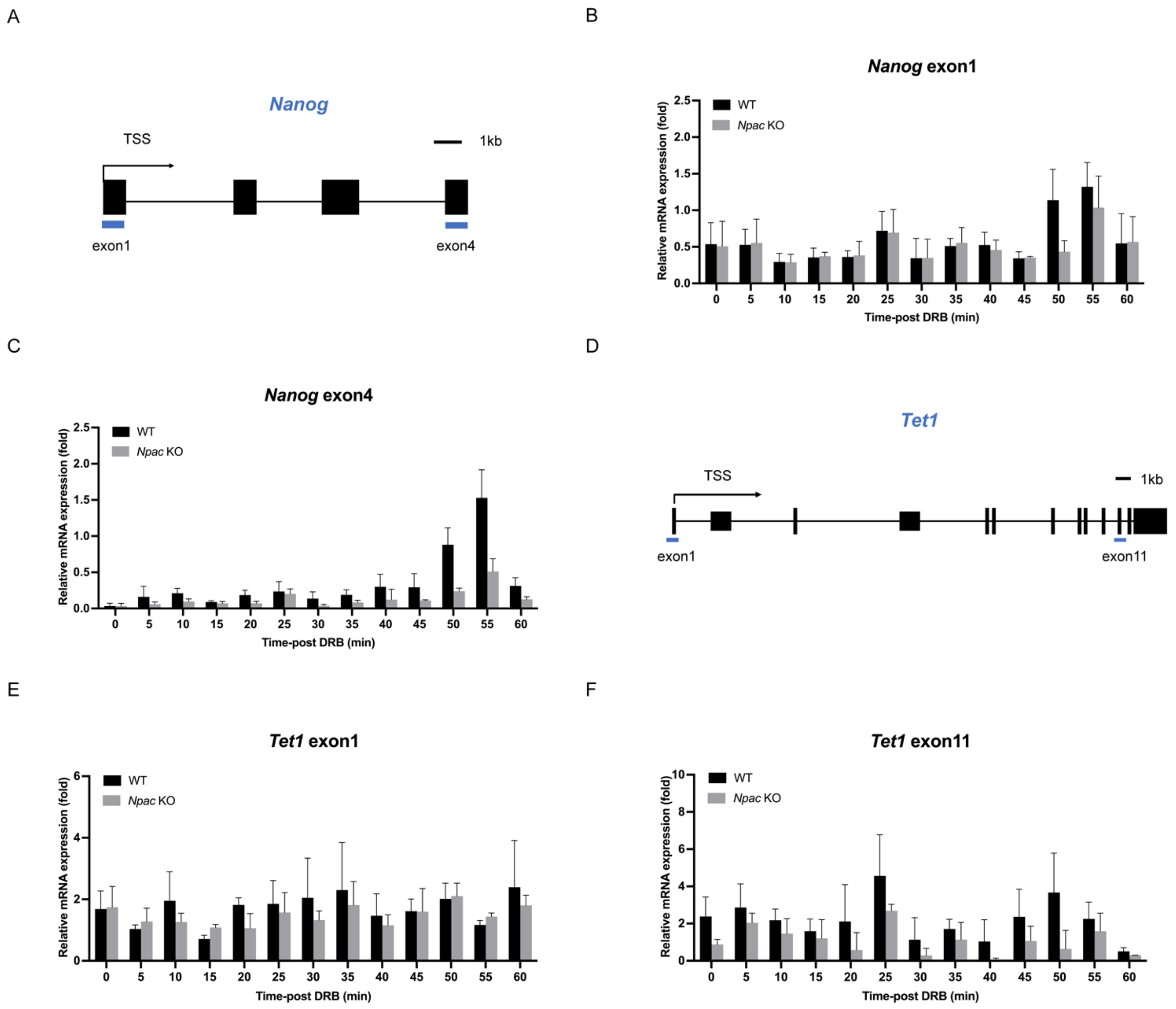

Figure S2. Transcription elongation recovery assay. (A) Regions analyzed on the *Nanog* gene are indicated in the figure. (B & C) Transcription rates of different regions of *Nanog* after *Npac* knockout are presented. (D) Regions analyzed on the *Tet1* gene are depicted in the figure. (E & F) Transcription rates of various regions of *Tet1* following *Npac* knockout are shown. Each panel represents the analyzed locations and the RNA levels released at different time points post DRB inhibition, as illustrated in the associated figure.

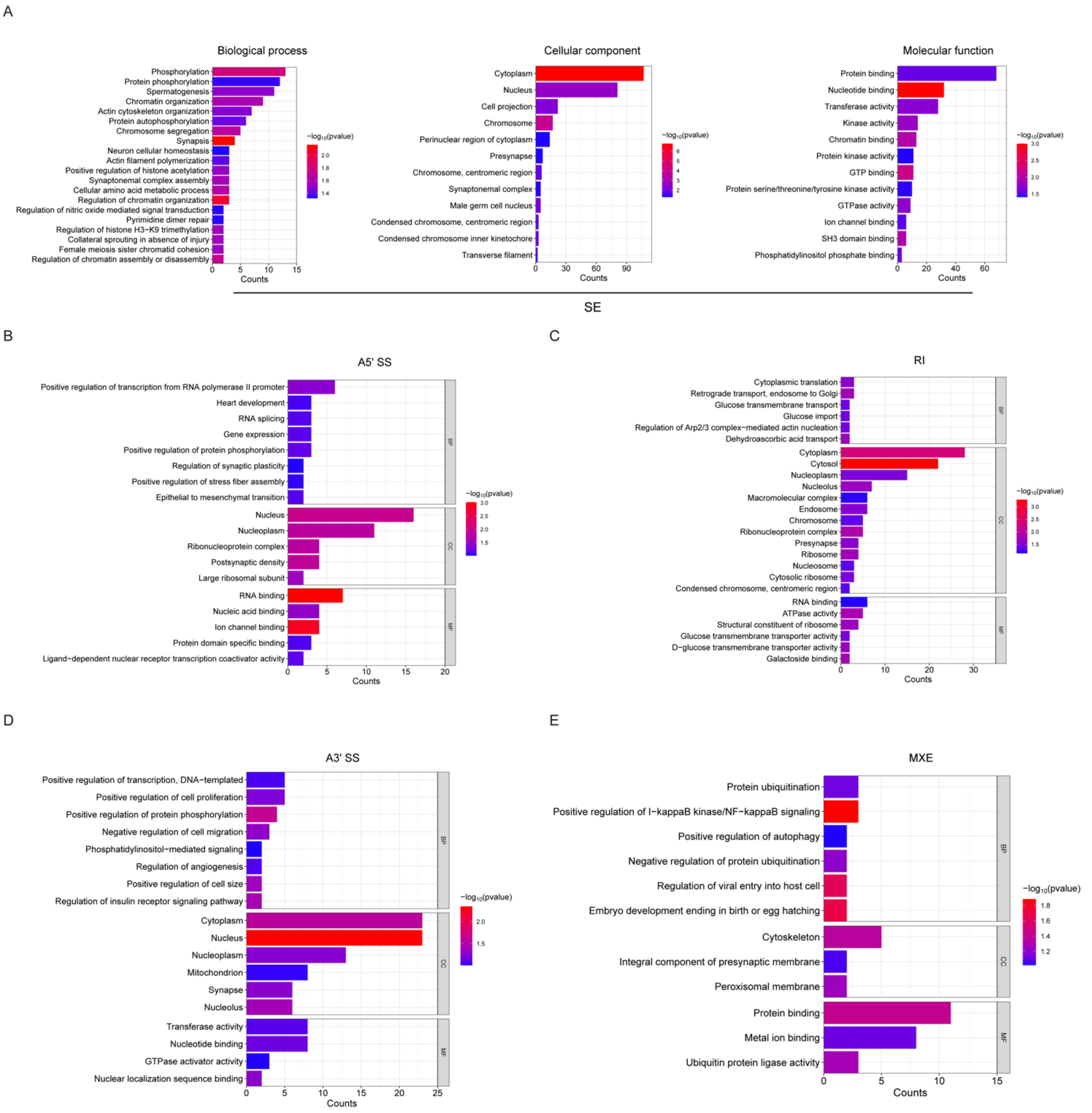

Figure S3. Gene Ontology (GO) analysis of differential alternative splicing events. The DAVID resource was utilized to analyze genes displaying skipped exons (SE), alternative 5' splice sites (A5'SS), retained introns (RI), alternative 3' splice sites (A3'SS), and mutually exclusive exons (MXE), focusing on their molecular functions, biological processes, and cellular components.

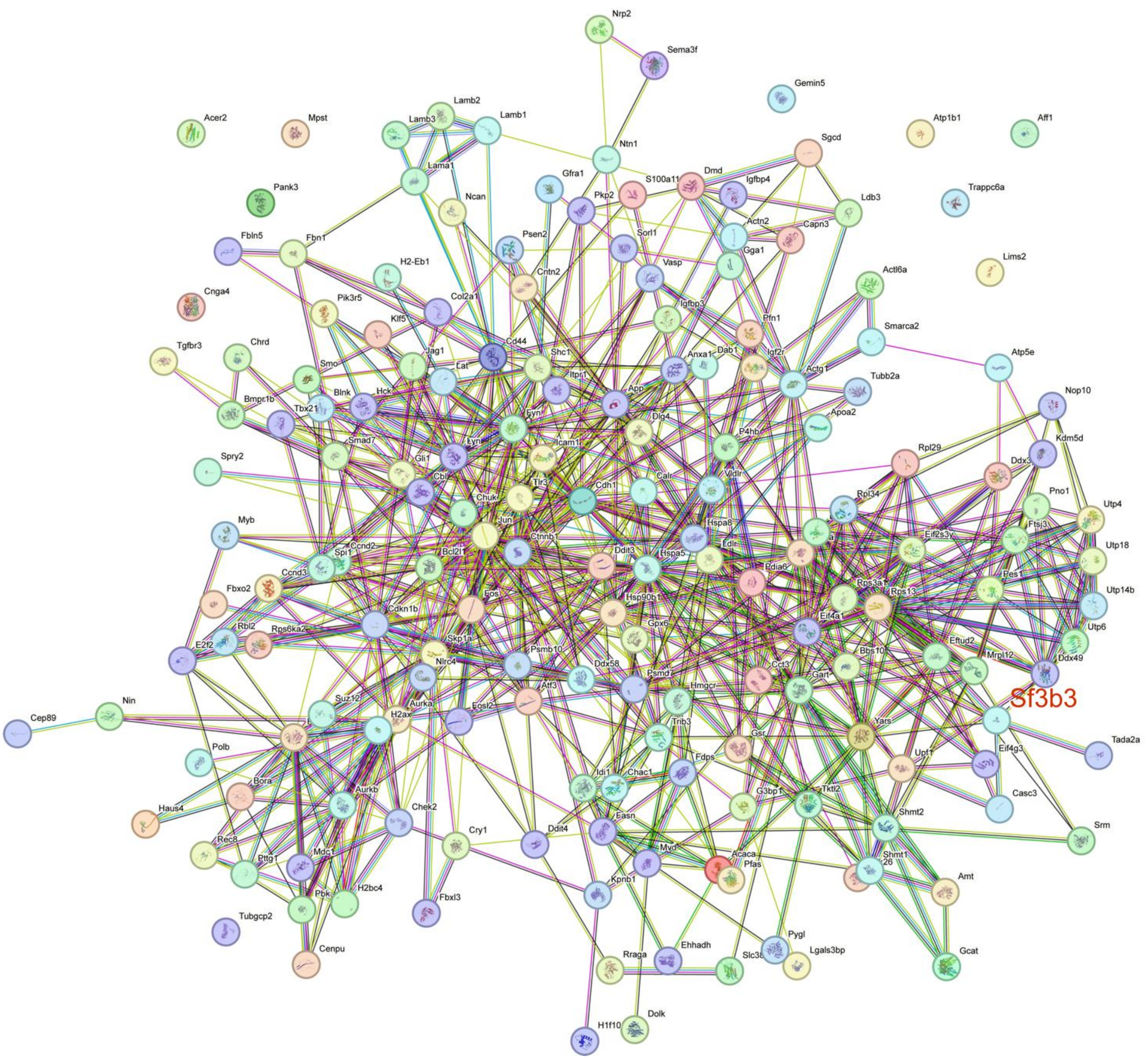

Figure S4. The differentially expressed genes were analyzed using the STRING database.

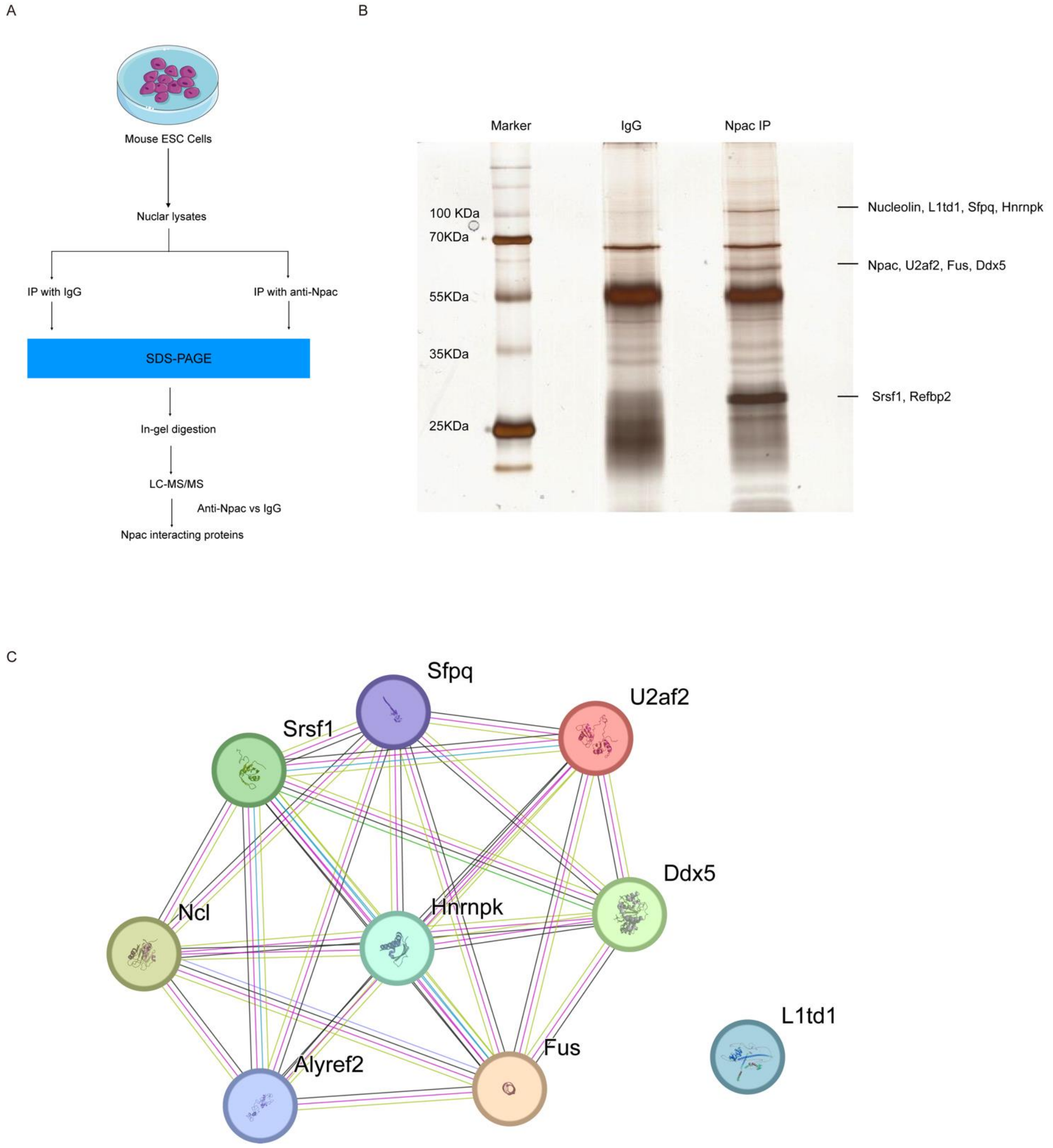

Figure S5. Proteins that interact with Npac. (A) The strategy and workflow for the identification of Npac interacting proteins in cells are illustrated. (B) To detect Npac-interacting proteins, cells underwent immunoprecipitation with an anti-Npac, juxtaposed with control IgG. (C) The STRING database facilitated the analysis of proteins associated with Npac. In this network, an edge signifies a previously established interaction between a pair of proteins.

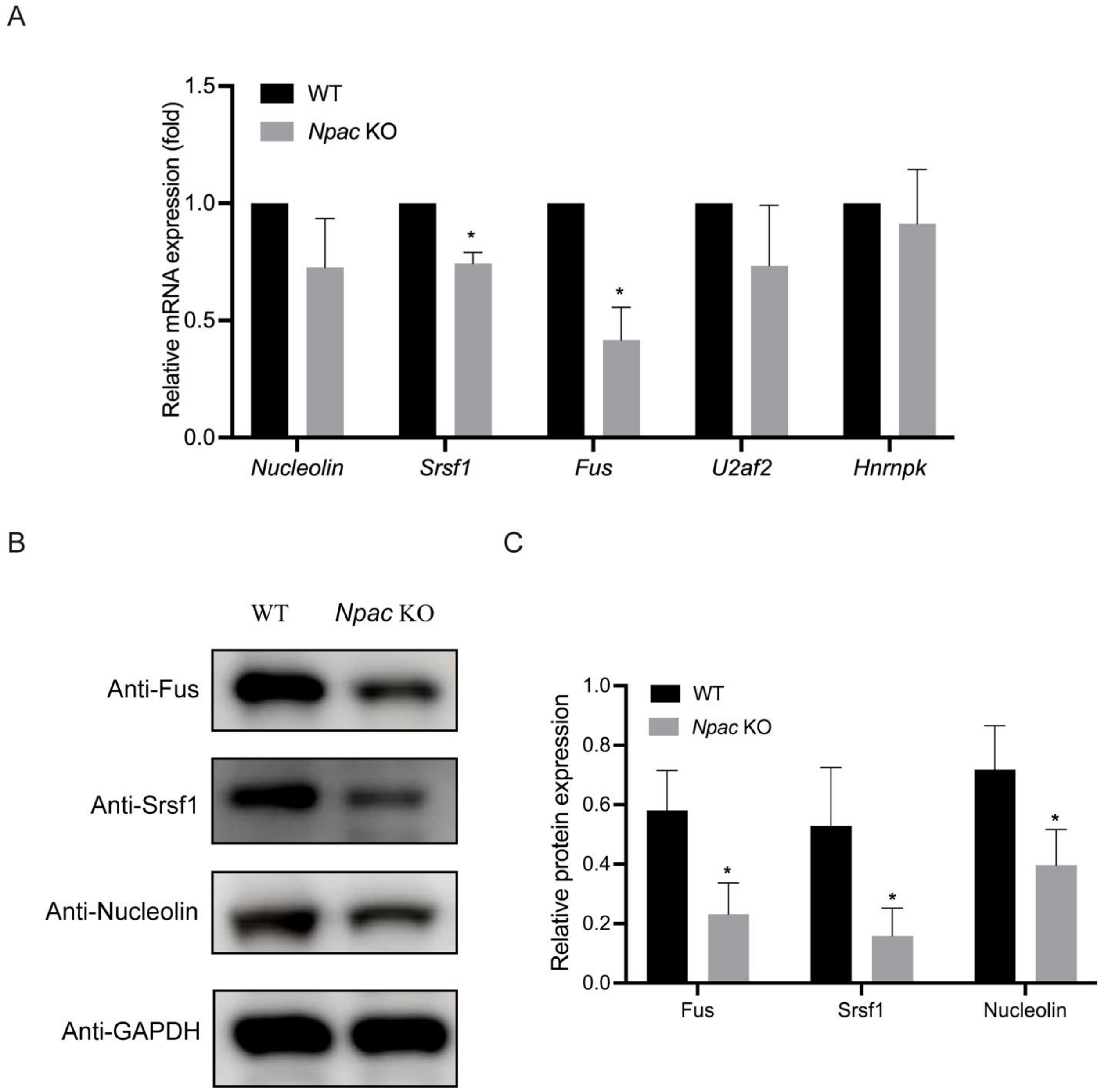

Figure S6. (A) The gene expression levels in E14 cells were quantitatively analyzed through RT-qPCR assays. (B) The presence and expression levels of the protein in E14 cells were assessed using Western blot analysis. (C) Subsequent protein quantification was conducted.

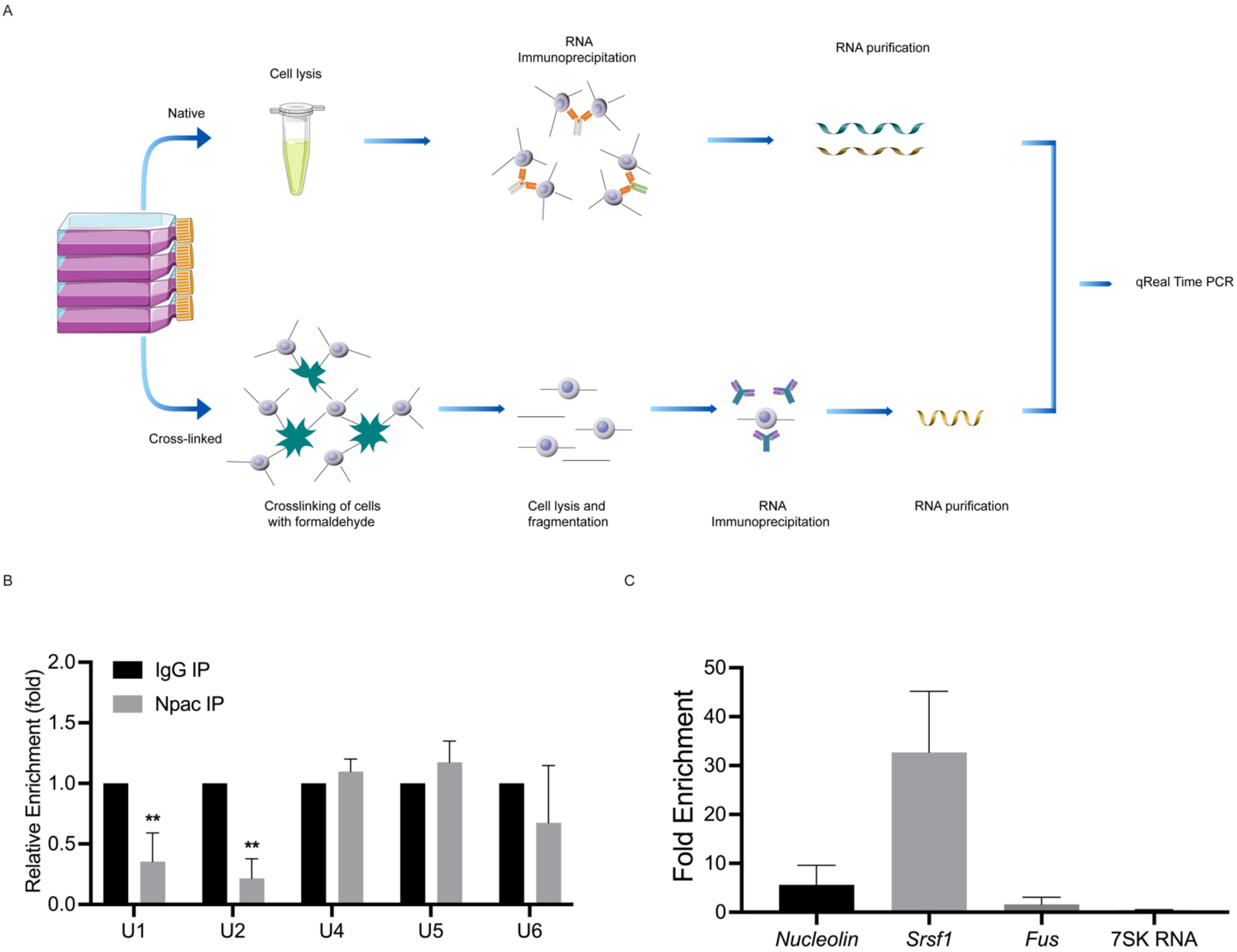

Figure S7. (A) Schematic summary of the RIP protocol. (B) Cross-linked RIP with Npac, or control IgG antibody. The relative of U SnRNA enrichment detected by RT-qPCR. (C) A native RIP assay coupled with RT-qPCR was conducted to confirm the interaction between Npac and Srsf1, Fus, and Nucleolin.

Supplementary Table S1. Sequences used in this study.

Gene name	Primer Sequence （5’-3’）
Nanog	Forward GGTTGAAGACTAGCAATGGTCTGA<br>Reverse TGCAATGGATGCTGGGATACTC
Sox2	Forward CCAGGAGAACCCCAAGATGCACAACT<br>Reverse AAGCCTCCGGGAAGCGTGTACTTATCCTT
Oct4	Forward TTGGGCTAGAGAAGGATGTGGTT<br>Reverse GGAAAAGGGACTGAGTAGAGTGTGG
Npac-1	Forward ACCACGTGGAAAGAAATGCT<br>Reverse CCTTGTGAGCATGGTAAGGC
Npac-2	Forward GAAACCACGTGGAAAGAAATGC<br>Reverse AGCTGTTCCACTTTGATCCAG
Nodal	Forward TCACCGTCCCCTCTGGCGTACATGTTGAG<br>Reverse CCCCTTGGCCTGGTGGAAAATGTCAATGG
Cdx2	Forward CGCAGAACTTTGTCAGTCCTCCGCAGTACC<br>Reverse GTATTCGGCGGGGCTGCTGTAGCCCATAGC
Bmp4	Forward GCCTAGCAAGAGCGCCGTCA<br>Reverse TCCGGGTACTCAAGCCCGGT
Foxa2	Forward CCTACGCCAACATGAACTCGATGA<br>Reverse GTAGAAAGGGAAGAGGTCCATGATCCACT
Hand1	Forward ATCACCACTCACACCCGCCG<br>Reverse CGGCAGGGAAATCTGGGGCA
Sox17	Forward TGAAAGGCGAGGTGGTGGCGAGTAG<br>Reverse CAACGCCTTCCAAGACTTGCCTAGCATCT
Gata6	Forward TGTGCAATGCATGCGGTCTCTACAGCA<br>Reverse TTCATAGCAAGTGGTCGAGGCACCC
Gata2	Forward GGCCTCTTCTTCTGCAGGGGGTAGTGTAG<br>Reverse GCACATAGGAGGGATAGGTGGGTATCGG
Rest	Forward CGGTGGCGGCGGAGACTAAA<br>Reverse GGCTCTTCGGAGGGATGGGC
Nestin	Forward AGAGGAAGAGCAGCAAGGCCATGAC<br>Reverse TCCCTGACTCTGCTCCTTCTTCTTCAT

Gfap	Forward CGGAGACGCATCACCTCTG<br>Reverse AGGGAGTGGAGGAGTCATTCG
Nkx2.5	Forward GAAGGCAGTGGAGCTGGACAAAGCCGAGA<br>Reverse GGAACCAGATCTTGACCTGCGTGGACGTG
Brachyury	Forward GAGCTGTGGCTGCGCTTCAAGGAGCTAAC<br>Reverse CCCCAGGTACCCACTCCCCGTTCACATA
Pax6	Forward GGGCGCAGACGGCATGTATGATAAA<br>Reverse AGTCGCATCTGAGCTTCATCCGAGTCTTC
Msx1	Forward ACAGAAAGAAATAGCACAGACCATAAGA<br>Reverse TTCTACCAAGTTCCAGAGGGACTTT
β-actin	Forward ATGACGATATCGCTGCGCTGGTC<br>Reverse GGCCCACGATGGAGGGGAATAC

Supplementary Table S2. Sequences used in this study.

Gene name	Primer Sequence （5’-3’）
Srsf6	Forward CGTGTACGAGCTCAACAGCAAG<br>Reverse CCAGAAGTTCTCCGACTGCTGTATC
Polh	Forward GCTTCTGTCATTGAAGTCCTTGG<br>Reverse GGTTAGCCTCTCCTCAAGTTCC
Hmces	Forward CTTCAGACAGCTCTGACAACAAGG<br>Reverse GGATTAGCTTCAGAGCTTCCTGAG
Mettl23	Forward CCAGCTCTTTCGGTTCCG<br>Reverse CAGCTTTGCCTGGTAGAGATC
Rnps1	Forward GTCCTGGTCTGAGAAGAGCG<br>Reverse GCGTCTCTTCCGAGTCTTATCTC
Atrx	Forward CTATCTGCGGTGACTACTAAGATCC<br>Reverse CAGAATTAAGTCCAGAACCACAGA
Rbpms2	Forward GTGGAGGTAACGAACGGAACC<br>Reverse GAAGAGCAGGTAGAGTTCTCTAGG
Pou2f1	Forward CAAGCCTTGTGACAAGCAGTAC<br>Reverse CTGTGTCGTGTTGGTCTCACTG
Ddb2	Forward GTTGATGCACAAGGCCAGTG<br>Reverse CTCCTTGCCATCTGTGCTCAACAG
Hsf5	Forward CTACTGCCTCTTCCTATGCACAC<br>Reverse CTTCGCTCTCTGTATCTAGCTGGAG

Supplementary Table S3. Sequences used in this study.

Gene name	Primer Sequence （5’-3’）

Nanog exon1	Forward GAGTTTTTTGGTTGTTGCCTAAAAC<br>Reverse TCAGTAGCAGACCCTTGTAAGCAAG
Nanog exon4	Forward TTTACATCTTTAGAGTCCCCTTTTC<br>Reverse TGGCTGCTCCAAGTTGGGTTGGTCC
Tet1 exon1	Forward GGTGGCCTCTGGCTTCTTTTTG<br>Reverse CTCCCCCATGTAATTCTACGC
Tet1 exon11	Forward ATTACACAAAGACAAATCAACC<br>Reverse GTGTTTCATATTGTATTTCATCAGA

Supplementary Table S4. Sequences used in this study.

Gene name	Primer Sequence （5'-3'）
U1	Forward TACTTACCTGGCAGGGGAGATA<br>Reverse CAGGGGAGAGCGCGAAC
U2	Forward ATCGCTTCTCGGCCTTTTGG<br>Reverse GGTGCACCGTTCCTGGAG
U4	Forward GCTTTGCGCAGTGGCAGTAT<br>Reverse TCTCCGTAGAGACTGTCAAAAATTG
U5	Forward CTGGTTTCTCTTCAGATCGT<br>Reverse GTTGGAGCAGAACCTCAAAAAATT
U6	Forward GTGCTCGCTTCGGCAGC<br>Reverse AAAAATATGGAACGCTTCACGAAT
Srsf1	Forward TACCGAGATGGCACTGGTGT<br>Reverse AGGCAGTTTCTCCCTCGTGA
Fus	Forward TCAATAAATTTGGTGGTCCTCGG<br>Reverse TGCTTGAAGTAATCAGCCACAG
Nucleolin	Forward TCCAAGTCCCACTCCCAACA<br>Reverse ATCTAATGCCCACGCCATCC
U2af2	Forward GATAGTGCCACAGGGCTCTC<br>Reverse TCACCAGCGTGGCATTCTT
Hnrnpk	Forward GTTGGGTTCAGTGCTGATGA<br>Reverse GATCACCATATGAGCCACGG
